# Ethnobotanic importance of plants used in pigeon-breeding in Eastern Spain

**DOI:** 10.1186/1746-4269-9-33

**Published:** 2013-05-20

**Authors:** Antonio Belda, Carolina Cortés, Victoriano Peiró

**Affiliations:** 1 Departmento Ciencias de la Tierra y Medio Ambiente, Campus San Vicente. Ap. 99-E03080, Alicante, Spain; 2 Departamento de Ecología, Alicante, Spain; 3 IMEM, Universidad de Alicante, Alicante, Spain

**Keywords:** Pigeon fancying, Pigeon breeders, Ethnobiological knowledge

## Abstract

**Background:**

The importance that birds of the *Columbidae* family have had throughout history is visible on the Mediterranean coast. Pigeon fancying is the art of breeding and training carrier pigeons and currently, several breeds exist. The sport of racing pigeons consists in covering a distance at maximum possible speed. However, pigeon breeding has another modality called “sport pigeon”, where several males follow a female. This study focusses on ethnobotanical knowledge of native and exotic plant species that are used for diet, breeding, stimulation, healing illnesses and staining the plumage of pigeons bred in captivity.

**Methods:**

Using semi-structured interviews, we gathered information about the different plant species traditionally used for pigeon-breeding in the region of Valencia. Background material on remedies for bird illnesses was gathered from folk botanical references, local books and journals.The plant species were collected in the study area, then identified in the laboratory using dichotomous keys and vouchered in the ABH (Herbarium of Alicante University). We used Excel ^®^ 2003 to perform a simple statistical analysis of the data collected.

**Results:**

We collected 56 species of plants (and one variety) that included 29 botanical families. The total number of species was made up of 35 cultivated and 21 wild plants. The most common were Gramineae (14 species), Leguminosae (6 species), and Compositae (4 species).

**Conclusions:**

Pigeon breeding is an immensely popular activity in Eastern Spain, and ethnobiological knowledge about breeding pigeons and caring for them is considerable. The names and traditional uses of plants depend on their geographical location, vernacular names serve as an intangible heritage. Feeding, environmental features, and genetic makeup of individuals are relevant aspects in the maintenance of avian health.

## Background

The importance that the birds of the Columbidae family have had throughout history is visible on the Mediterranean coast where Roman legions passed. This coast had many towers with pigeons that carried important news. Engravings, reliefs and coins demonstrate the importance that these birds had for the Romans. The Egyptians and Phoenicians used pigeons for communication, although it was the Arabs who were responsible for the introduction of several breeds into Spain and the Balearic islands. It is more than likely that the Normans introduced this kind of Mediterranean pigeon to England, called “carrier” pigeon in the English countryside.The use of carrier pigeons was lost in the Middle Ages or was limited. Belgium was considered the greatest diffuser of modern pigeon breeding, as it was in this region where the modern kind of carrier pigeon was developed [1].

In the 1840s and 1850s, the rapid growth of the telegraph led to a decline in the use of pigeons for sending messages and some birds used by merchant and press agencies came onto the open market. This led to the growth of pigeon racing amongst individuals. In early 1855, Charles Darwin and his family spent several weeks in London. This was when Darwin may have noticed common pigeons foraging for oats from spilt horse feed on one of his walks and perhaps this was what inspired him to prove that all fancy pigeons are descended from the common pigeon, also known as the Rock Dove (*Columbia livia* Gmelin). This particular research would also help him with his theories towards the 'Origin of Species' [2].

It was in the last third of the nineteenth century that pigeon racing became firmly established in working-class culture. In 1886, King Leopold II of the Belgians gave racing pigeons to the British Royal Family as a gift and they were used to start a racing loft on the Sandringham Estate. Pigeons played an important role in both World Wars and were used by the military as messengers due to their homing abilities. Pigeons from the royal loft were even used as carrier pigeons during the First and Second World Wars, with one bird winning the Dicken Medal for Gallantry for its role in reporting a lost aircraft in 1940 [3].

Pigeon racing before the 1890s was largely a localized, even introspective, sport of short-distance competitions. The working class world of short-distance racing continued up until the Second World War and beyond, although it was in decline and being replaced from the end of the nineteenth century by the more formalized and socially diverse long-distance racing. Nowadays, apart from pigeon racing, some men have passed on their love of pigeons to their children and it has become a shared hobby, an opportunity for fathers and children to spend free time with each other [4].

Pigeon fancying is the art of breeding and training carrier pigeons and currently, several breeds of carrier pigeons exist. The sport of racing pigeons consists in covering a distance at maximum possible speed. However, pigeon breeding has another modality called “sport pigeon”, where several males follow a female. The country with most licences is China (300,000 in 2005). There were 23,100 licences in Spain in 2012, of which 8,700 were in the Valencian region. The total number of pigeons in this region is approximately 261,000 [5].

*Columbia livia* Gmelin is native to Eurasia and North Africa. Its natural habitat is rocky cliffs, or coasts and deserts, although it is considered to be a pest in cities. The female lays two eggs and both sexes incubate. Reproduction takes place all year round [6].

This species is not considered to be in any kind of risk category [7], although its wild population has declined [8].

**Figure 1:**
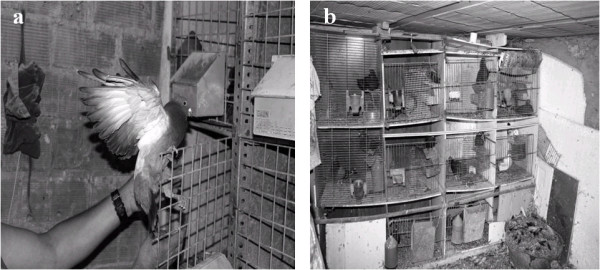
**Pigeons bred in captivity: a) Pigeon. b**) Traditional dovecotes.

Previous ecological studies exist about reproduction and methods of *Columba livia* control in Europe [9] and about their diet. According to the bibliography consulted, *Columba livia* feeds in fields and herbaceous zones, gathering seeds, buds, berries and small invertebrates [10]. In Spain, there is little ethnobotanical bibliography available about the diet and care of Columbidae. However this kind of study does exist for Fringillidae [11]. This study focuses on ethnobotanical knowledge of native and exotic plant species that are used for diet, breeding, stimulation, healing illnesses and staining the plumage of pigeons bred in captivity (Figure 1). Therefore, the transmission of traditional knowledge related to the role that plants play in this traditional activity and their relation with human medicine is necessary [12].

## Materials and methods

### Study area

**Figure 2:**
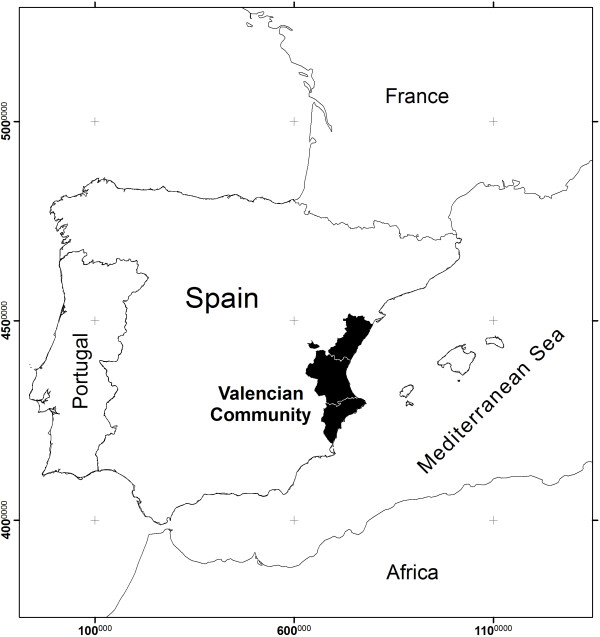
Study area.

The Region of Valencia (Comunidad Valenciana, in Spanish) is an autonomous community located on the eastern coast of the Iberian Peninsula (Figure 2). The total area is 2,325,874 ha and it is formed by three provinces, Castellón (667,869 ha), Valencia (1,076,204 ha) and Alicante (581,901ha). The total number of 536 municipalities is grouped into 34 districts, with a total population of 3,857,234 inhabitants [13]. The official languages of this region are Spanish and Valencian, a Catalan dialect.

The latitude of the Valencian territory lies between 37º 51’ and 40º 47’ North. The geophysical environment is diverse, there are plains, valleys, plateaus, etc., and differences in altitude that range from sea level to 1800m. The relief is a principal factor that explains the variety in climate. Average daily solar radiation is about 17 MJ (megajoules).

The climate is dry in summer thanks to the widespread influence of the Azores anticyclone, whereas the winters are mild, although there is frost and sometimes snow in zones in the interior. In general, the most important rains fall in autumn, although the spring rains are very important in some western regions, and this spring rainfall means a recovery of heat and humidity. Summer and winter are not dry seasons, due to the Iberian mountainous zones, where frequent summer storms are registered. The irregular character of rainfall throughout the year, especially in coastal zones, is due to the great frequency of torrential thunderstorms in the Levant [14].

The vegetation shows 4 large altitudinal belts that form bioclimatic floors: Termomediterranean (17–19°C), Mesomediterranean (13–17°C), Supramediterranean (13-17°C) and Oromediterranean (4–8°C). Most forests in the territory are Mediterranean pine groves formed by *Pinus halepensis*, *P*. *nigra* subsp. *salzmannii* or *P*. *pinaster*. We also find juniper forests, with a more open structure [15,16]. In general, pines (87%) dominate the composition of the Valencian woodland. These trees have adapted well to the current climate, spontaneously or by human intervention, to arrive at the verdant vegetation (13%) characteristic of the Valencian vegetation climax with a slow reproduction process. The most important Valencian woodlands are located in the mountains and interior plateaus of Valencia and Castellón, and are less common in Alicante [15].

The climatic formations of the coastal dunes lack the habitual arboreal type found in other similar zones of the Mediterranean, probably as a consequence of the unfavourable water regime and overexploitation suffered in the past [17].

### Methods: Interviews, field and literature information

A total of 26 municipalities were studied in the three provinces. We wanted to emphasize the ethnobotanical importance of local variations of plant names and the different applications of these species. All interviewees were rural people, who came from a variety of socioeconomic backgrounds, and were selected because of their long-term participation in pigeon breeding.

Using semi-structured interviews, we gathered information about the different plant species traditionally used for pigeon-breeding in the region of Valencia. This information was further verified via participant observation in the field, where fanciers were observed preparing for and participating in competitions and the different species of plants used by bird breeders were identified. Support material on remedies for bird illnesses was gathered from folk botanical references [18,19], local books [16] and journals [20]. The plant species were collected in the study area, then identified in the laboratory using dichotomous keys [21], and vouchered in the ABH (Herbarium of Alicante University). We used Excel ^®^ 2003 to perform a simple statistical analysis of the data collected; specifically, we calculated the relative frequency of citation (RFC), i.e., the percentage of interviewees that mentioned the use of the species (Table 1). In addition, we calculated a cultural importance index (CI), where each addend is a measure of the relative importance of each plant use category. The cultural importance index is due to an interest in detailing the specific uses of plants that best reflect the cultural aspects of plant use [22].

**Table 1:** **List of plant species and their uses in pigeon**-**breeding in the Valencian region** (**Southeast Spain**)

**Scientific name**	**Herbarium Voucher (ABH)**	**Family**	**Spanish name**	**Catalan name**	**Uses**^**a**^	**Frequency of citation**	**CI**	**Type**^**b**^	**Plant part used**^**c**^
*Allium sativum *L.	Seen alive	Alliaceae	Ajo	all	6	73.52	1.120	C	A,R
*Arundo donax *L.	32085	Gramineae	Caña	canya	3	0.98	0.009	W	L, S, F
*Avena sativa *L.	10488	Gramineae	Avena	avena, civà, civada, cogula	1,2,4	64.70	0.881	C	Fr,Se
*Beta vulgaris *L.	10652	Chenopodiaceae	Acelga	bledes	1,2,4	58.82	0.117	C	L
*Beta vulgaris *var.*cycla *(L.) K.Koch	Seen alive	Chenopodiaceae	Remolacha	remolatxa	7	1.96	0.019	C	L
*Brachypodium retusum *(Pers.) Beauv.	31248	Gramineae	lastón, fenal	fenàs, llistò	3	34.31	0.245	W	A
*Brassica napus *L.	39373	Cruciferae	colza, nabina	nap	1,2,4	31.37	0.586	C	A
*Cannabis sativa *L.	32225	Cannabaceae	Cáñamo	cànem	5	78.43	0.784	C	R,S,Se
*Carthamus tinctorius *L.	3894	Compositae	alazor, cártamo	càrtam, safrà dels pobres	6	39.21	0.725	C	Fr
*Chelidonium majus *L.	18328	Papaveraceae	hierba verrugera	herba berrugera	6	2.94	0.035	W	L, S
*Cicer arietinum *L.	17633	Leguminosae	Garbanzo	cigró	1,4,6	3.92	0.048	C	Fr
*Citrus limon *L. Burm. Fil.	34528	Rutaceae	Limón	llima	6	37.25	0.372	C	Fr
*Citrus sinensis *Osbeck	41947	Rutaceae	Naranjo	taronger	6	4.90	0.049	C	Fr
*Crocus sativus *L.	5207	Iridaceae	Azafrán	safrà	6	0.98	0.009	C	F
*Daphne gnidium *L.	10830	Thymelaeaceae	Torvisco	matapoll	6	5.88	0.058	W	L, S
*Daucus carota *L.	33104	Umbelliferae	Carlota	carlota	1,2,4	68.62	0.841	C	R
*Equisetum hyemale *L.	41037	Equisetaceae	cola de caballo, rabo de caballo	cua de cavall	3	2.94	0.029	W	S
*Ficus carica *L.	40887	Moraceae	Higuera	figera	1,2,4	1.96	0.057	W	Fr
*Foeniculum vulgare *Miller.	23129	Umbelliferae	Hinojo	fenoll,fonoll	3	32.35	0.323	W	F,Fr,L
*Helianthus annus *L.	5220	Compositae	pipa, girasol	pipa, gira-sol	1,2,4	76.47	1.557	C	F, Fr
*Hypericum perforatum *L.	18965	Guttiferae	hipérico, rubio perico	herba de Sant Joan, pericó	6	24.50	0.255	W	F
*Hordeum vulgare *L.	10648	Gramineae	Cebada	ordi	1,2,4	89.21	1.028	C	Fr
*Lactuca sativa *L.	Seen alive	Compositae	ensalada, lechuga	ensalada, ansalà, encisam, lletuga	1,2,4	65.68	0.872	C	F, Fr, L
*Laurus nobilis *L.	43242	Lauraceae	Laurel	llorer	6	1.96	0.019	C	L
*Lens culinaris *Medik.	35306	Leguminosae	Lenteja	llentilla	1,2,4	90.19	2.605	C	Se
*Lygeum spartum *L.	8128	Gramineae	Albardín	espart bord, almasset	3	2.94	0.029	W	L
*Malus domestica *(Borkh.) Borkh.	37495	Rosaceae	manzano	pomera	6	29.41	0.267	C	Se
*Matricaria chamomilla *L.	11812	Compositae	manzanilla	camamilla	6,7	36.27	0.538	C	A
*Medicago sativa *L.	37510	Leguminosae	alfalfa,mielga	alfals	1,2,4	24.50	0.450	W	A
*Nicotiana tabacum *L.	4391	Solanaceae	tabaco	tabac, tabaquera	6	0.98	0.009	C	L
*Origanum vulgare *L.	22141	Labiatae	orégano	orenga	1,2,4	53.92	0.567	C	L
*Oryza sativa *L.	14955	Gramineae	arroz	arròs	1,2,4	92.15	2.685	C	Se
*Phalaris canariensis *L.	Seen alive	Gramineae	alpiste	alpiste	1,2,4	90.19	2.419	C	Se
*Piper nigrum *L.	Seen alive	Piperaceae	pimienta negra	pebre negre	6	3.92	0.039	C	Se
*Phragmites australis *(Cav.) Steudel	40289	Gramineae	carrizo	carrís, senill	3	2.94	0.029	W	L, S
*Pinus halepensis *Miller	37506	Pinaceae	pino carrasco	pi, pi carrasco, pi blanc	3,8	8.82	0.107	W	L, Fr, S
*Pisum sativum *L.	7069	Leguminosae	guisante	pésol	1,2,4	93.13	2.744	C	Fr
*Prunus dulcis *(Mill.) D.A.Webb	12672	Rosaceae	almendro	ametler	6	4.90	0.042	C	Fr
*Portulaca oleracea *L.	46140	Portulacaceae	verdolaga	verdolaga	1,6	5.88	0.176	W	A
*Punica granatum *L.	46140	Punicaceae	granado	magraner	8	14.70	0.147	C	Fr
*Rhamnus alaternus *L.	7260	Rhamnaceae	aladierna, alaterno	aladern	6	6.86	0.068	W	F
*Ricinus communis *L.	36562	Euphorbiaceae	ricino	ricí	6	2.94	0.029	W	F, R
*Rubia peregrina *L.	6777	Rubiaceae	rubia roja,rubia tintorera	rosa tintorera	7	17.64	0.176	W	Ro
*Ruta chalepensis *L.	1484	Rutaceae	ruda, ruda silvestre	ruda	6	7.84	0.078	W	F
*Secale cereale *L.	10643	Gramineae	centeno	ségol	1,2,4	85.29	2.449	C	Se
*Sedum sediforme *(Jacq.) Pau	44375	Crassulaceae	uvas de pastor,uva de gato	raïmet de bruïxa	1,4	24.50	0.245	W	L
*Sesamum indicum *L.	40261	Pedaliaceae	sésamo, ajónjoli	sèsam	1,2,4	55.88	1.636	C	Se
*Sonchus oleraceus *L.	47365	Compositae	cerraja	llicsó, llicsó d'ase	6	4.90	0.049	W	F, Fr, L
*Sonchus tenerrimus *L.	37483	Compositae	cerraja	llicsó de perdiu, llicsó de cadernera	6	3.92	0.039	W	F, Fr, L
*Sorghum halepense *(L.) Pers.	37515	Gramineae	sorgo	canyota, canyot	1,2,4	100.00	2.617	C	Se
*Stipa tenacissima *L.	3298	Gramineae	esparto	espart, aspart	3	53.92	0.539	C	A
*Triticum aestivum *L.	40147	Gramineae	trigo, trigo candeal	blat	1,2,4	100.00	2.586	C	Fr
*Urtica dioica *L.	40147	Urticaceae	ortigas	ortigues	3,5,6	65.68	1.312	W	F,Fr,L
*Vicia ervilia *L.	55729	Leguminosae	yero	erb, évol, edro	1,2,4	83.33	1.626	C	Se
*Vicia sativa *L.	38937	Leguminosae	veza, garrobilla	veça	1,2,4	76.47	2.008	C	Fr
*Zea mays *L.	14049	Gramineae	maíz	dacsa, panís	1,2,4	100.00	2.773	C	Fr

^a ^Uses: 1, plants used for adult diet; 2, plants used for juvenile diet; 3, plants used in the composition of dovecotes; 4, mixtures used according to the seasons to favour the moult, mating, etc.; 5, plants used to stimulate mating; 6, cure diseases; 7, plants used for dyeing.

^b ^Type: *W *wild, *C *cultivated.

^c ^Plant part used: *A *whole plant, *F *flowers, *Fr* fruits, *L *leaves, *R *resin or sap, *Ro *roots, *S* stems, *Se *seeds.

## Results and discussion

## Results

We collected 56 species of plants (and one variety) that included 29 botanical families. The total number of species was made up of 34 cultivated and 22 wild plants. The most common were Gramineae (14 species), Leguminosae (6 species), and Compositae (4 species). We present the scientific names of these plant species, voucher registers, families, vernacular names, main uses, their citation frequency, cultural importance, whether the species is wild or cultivated in the Valencian region and the plant part used (Table 1). The data from the different associations of the Valencian region, and our study, inform us that pigeon fanciers are mainly men.

The most frequently reported species include *Sorghum halepense*, *Triticum aestivum*, *Zea mays*, all of which are mentioned by 100% of the informants and *Pisum sativum* (93.13%). The least common species include *Arundo donax*, *Crocus sativus* and *Nicotiana tabacum*.

Besides plants, the Valencian breeders use other substances (natural oils extracted from plants or animals, minerals, etc.) that are detailed based on our results.

The major categories and direct applications of these species as employed by bird hunters and breeders are as follows: 1) Plants used for diet of adult pigeons; 2) Diet for juvenile pigeons; 3) The composition of dovecotes 4) Mixtures to favour the moult; 5) To stimulate mating; 6) To cure diseases, including vulnerary to stop haemorrhages, or plants with antibacterial properties, to promote moulting, disinfectant functions, and which can host beneficial probiotic bacteria (e.g. sap of *Allium sativum*); 7) Plants used to dye plumage.

The species least used according to our study are 1) *Crocus sativus* for dyeing (CI=0.009); 2) *Arundo donax* in composition of dovecotes (CI=0.009); 3) *Nicotiana tabacum* for curing diseases (CI=0.009).

Approximately, 71.4% of the interviewees used natural oils. The most important of these are *Prunus dulcis* oil to prevent constipation, cod liver oil to favour the moult (especially in summer), and to stimulate growth in young pigeons, *Ricinus communis* oil as a purgative and to cure coughs, *Hypericum perforatum* oil to cure external injuries or *Allium sativum* oil as a natural antibiotic to recover pigeons’ strength after taking part in a competition and to stimulate the moult. These are frequently used by pigeon keepers. Breeders told us how they obtain natural oils; in the case of *P*. *dulcis*, the fruit is peeled and compressed, separating the fatty oils. The resultant mixture is macerated in water and distilled. Later the hydrocyanic acid is removed as it is toxic. A yellow and sweet liquid with an intense aroma is obtained. Cod-liver oil is obtained by cold-pressing the liver. *Ricinus communis* oil is obtained using an expression process. The *Allium sativum* oil is produced by maceration of several cloves of *Allium sativum* in olive oil for 30–40 days. The flowers of *Hypericum perforatum* are also macerated for 40 days. Nowadays, most pigeon-breeders buy these oils in pet shops; however, we have recovered the traditional fabrication processes for some of them.

Other natural remedies are *Urtica dioca* infusions, *Cannabis sativa* seeds or legumes such as *Lens culinaris*, or *Pisum sativum* to stimulate mating; *Cicer arietinum* with water, *Malus domestica* vinegar or *Allium sativum*, are used as probiotics. In addition, barley water is employed for refreshment and *Origanum vulgare* extract to minimize intestinal settling of undesirable bacteria. *Piper nigrum* and *Ruta chalepensis* are utilized to treat hepatic diseases. Likewise, branches of *Laurus nobilis* are used to prevent fleas, nettles to provide minerals for fractured bones, *Nicotiana tabacum* ash is used to stop bleeding and *Allium sativum* to prevent moths. Furthermore, *Punica granatum* peel, *Rubia peregrine*, *Pinus halepensis*, *Matricaria chamomilla*, *Crocus sativus* or *Beta vulgaris* var. *cycla* are employed to dye the plumage of pigeons. *Stipa tenacissima* grass and *Urtica dioca* are used in the construction of dovecotes. Nowadays, the use of chemical products is also important for the health care of pigeons, for example, medicines such as Metronidazol are used for the treatment of trichomonas, Clazuril for avian coccidiosis, and furazolidona is mainly used for salmonellosis. Calcium hyposulphite and vitamin C and D_3_ are used to stimulate muscles before and after competition, and one drop of hydrochloric acid drop in a litre of water or orange juice serves to cure diarrhoea. Furthermore, copper sulphate is employed to cure warts, iodine is used to cure mange and rheumatism in legs and injuries and bicarbonate for liver disease.

We carried out a total of 102 oral interviews. All interviewees were male, 48.96% were Spanish speakers and 53.04% spoke Valencian, and they ranged in age from 27–83 (mean of 53 years). The average age at which their interest in breeding domestic pigeons began was 21. Approximately, fifty-two per cent (51.9%) of the interviewees take part in competitions, and the average monthly expense dedicated to the purchase of seeds in shops is 22.44 € per month. The average monthly expense dedicated to feeding per pigeon is 0.53 €.

## Discussion

This study reveals that pigeon breeders in Iberia have a high degree of plant-bird ethnobiological knowledge, which is principally reflected by their identification and application of useful plant species. Valencian breeders know that it is necessary to take diverse factors into account in order to elaborate a diet adapted for pigeons, such as the biological diversity of a breed or species, the season of the year, digestibility of food and the category of the pigeons. These categories are qualified as pigeon, juvenile, adult, adult in reproduction, bird at the moment of the moult, bird under conditions of intense exercise, as in the case of competition pigeons (racing pigeons and sport pigeons) and sick birds. Names and traditional uses vary depending on geographical location, and vernacular names serve as intangible heritage. Our bibliographical review confirms some of the traditional uses of local plants identified in the study area [17,19].

The results of studies about ecology and behaviour of *Columba livia* have shown different types of seeds in the diet of *Columba livia* in western Paleartic regions [23]. The most common botanical families in this article are Leguminosae, and Compositae. Another study analyzing the food of a feral population of *Columba livia* in Central Europe [24] showed that seeds of cereals form 68% of its weight, seeds of other cultured herbs 17.5%, garbage 10.5%, seeds of woody species 1%, seeds of wild herbs 1%, green parts of herbs 0.5%, indigestible components 1.4%, and animal components 0.1%.

The pigeon fanciers from the Valencian region showed a slight preference for using cultivated species. In general, our results coincide with the results of these articles and with the description of Segrelles, who described in his book that pigeon diet is basically made up of seeds and fruits [25], with cereals and vegetables predominating. The most common families in our study are Gramineae, Leguminosae and Compositae, whose fruits and seeds are mainly used for pigeon diet.

Similarly, as for the feeding and nutrition of wild birds in captivity, environmental features and the genetic makeup of individuals are key aspects in the maintenance of avian health. Valencian breeders know which conditions are optimal for maintaining this group of birds in captivity, for instance, feeding the doves with species of plants that facilitate thermoregulation, due to high concentrations of lipids and proteins [26], low blood pressure or activate the appetite and help digestion [25]. Apart from the wide knowledge that Valencian breeders demonstrate about the plants used for the care of their pigeons, they also use a series of traditional remedies. Some of these are ground mollusc shells to favour the fixation of calcium, lard and sulphur to treat mange, copper sulphate for warts, iodine for wounds and rheumatism in legs, nitrous acid to disinfect dovecotes, etc. Moreover, pigeons play an important role in the conservation of predatory birds, due to the fact that most of them prey on pigeons [27]. Regarding the cultural importance index, *Zea mays* (CI= 2.773), *Pisum sativum* (CI=2.744), *Oryza sativa* (CI=2.685), *Sorghum halepense* (CI=2.617), *Lens culinaris* (CI=2.605) and *Phalaris canariensis* (CI= 2.419) have the greatest CI. This is because these seeds, both cultivated and wild, are the ones that are used most in feed mixes for different species of pigeons kept in captivity. The lowest CI was obtained for *Crocus sativus* (CI= 0.009), *Arundo donax* (CI= 0.009) and *Nicotiana tabacum* (CI=0.009). The use of *Crocus sativus* as a dye for plumage has been taken over by commercial chemical dyes. *Arundo donax* has fallen into disuse because there are other species more accessible for breeders such as *Stipa tenacissima* (CI= 0.539), *Foeniculum vulgare* (CI= 0.323) or *Brachypodium retusum* (CI=0.245). The ashes of *Nicotiana tabacum* are useful for stopping haemorrhages. This is a use that we have found in few pigeon-breeders but it is curious and relevant. These reasons explain the lower CI values in the table. Many of the plants mentioned in this study for the care of pigeons are also used in human medicine, for example, *Allium sativum* is thermoregulatory, *Urtica dioica* is employed to reduce inflammations or to regulate anaemia, *Citrus limon* for injuries or as a depurative, *Sorghum halepense* for toothache, etc. [12]. Many of the traditional uses of plants used in the cure of animals are also beneficial for humans.

## Conclusions

Pigeon breeding is an immensely popular activity in Eastern Spain, and ethnobiological knowledge about breeding pigeons and caring for them is considerable. However, our observations suggest that traditional knowledge about feeding and healing birds with plants is being lost, due to the use of new commercial products. The names and traditional uses of plants depend on their geographical location, vernacular names serve as an intangible heritage. The maintenance of the pigeon breeding tradition is mainly due to the transmission of knowledge between members of the same family. The lack of this transfer of information would mean an important loss of ethnobotanical knowledge in the care of pigeons. There are also people that work in pigeon breeding as a vocation, but this is less common. Feeding, environmental features, and genetic makeup of individuals are relevant aspects in the maintenance of avian health. Our hope is that this research will highlight a longstanding but until now little-known ethnobotanical tradition and that it will assist in the recovery of almost forgotten uses and traditions.

## Competing interests

The authors declare that we have no competing interests.

## Authors' contributions

AB carried out bibliographical search, drafting of the manuscript and final review of the document. CC carried out accomplishment of interviews, gathered from information, drafting the manuscript and final review of the document. VP carried out supervisión of the study and final review of the document. All authors read and approved the final manuscript.
